# Performance level discriminative validity of agility tests in youth tennis players

**DOI:** 10.3389/fspor.2025.1486777

**Published:** 2025-05-09

**Authors:** Goran Munivrana, Goran Jelaska, Mario Tomljanović

**Affiliations:** ^1^ Faculty of Kinesiology, University of Split, Split, Croatia; ^2^ Virovitica County Hospital, Virovitica, Croatia

**Keywords:** racket sports, change of direction, performance evaluation, measurement characteristics, competitive success

## Abstract

**Introduction:**

Various studies have shown that the ability to change the direction of movement quickly plays an important role in achieving top performance in tennis. The main goal of this study was to compare different generic and tennis-specific agility tests to determine whether and to what extent they can differentiate youth tennis players in terms of their competitive success and can be used as a tool to identify talented players in youth tennis categories.

**Methods:**

Thirty-three youth tennis players took part in the tests, 21 boys and 12 girls (11.05 ± 0.59 years), all of whom competed at national level in the U12 category and were divided into three different performance categories. Five validated agility tests covering three different test types (generic pre-planned CODs/tennis-specific pre-planned CODs/tennis-specific reactive tests) were selected to determine whether the type of agility tests used makes a difference in predicting the future competitive success of youth tennis players.

**Results:**

Both intra-subject and inter-subject reliability proved to be high for all agility tests used (Cα .87-.97; ICC .83-.94). The results also demonstrated the construct validity of the test battery used, as a significant latent dimension was extracted and all tests were projected fairly evenly onto the common factor. The between-subjects ANOVA showed that the results of the different agility tests can successfully differentiate young tennis players in terms of their competitive performance. The players who belonged to a higher performance level achieved better results in all agility tests used. However, the differences were only significant between players with “high” (1st) and “low” (3rd) performance levels in all the tests used, and additionally between players with “average” (2nd) and “low” (3rd) performance levels in the three tennis-specific agility tests (*p* < .05).

**Discussion:**

The results of the study suggest that agility tests have the potential to discriminate between different quality levels youth tennis players, regardless of which type of test (generic/tennis specific pre-planned/tennis specific reactive) is used.

## Introduction

Change-of-direction (COD) ability and agility as a whole, play an essential role in many sports, especially sports games. As noted in the paper of Inglis and Bird (1) agility has traditionally been defined as “the ability to change direction quickly and precisely” (2–5) or as “the ability to decelerate, reverse, or change movement direction and accelerate again” (6).

These definitions and views on agility do not take into account the fact that most directional changes in sport occur in response to a sport-specific stimulus. As suggested by Sheppard and Young (7), a definition of agility should consider not only physical and technical skills, but also cognitive processes. However, agility is usually trained and tested with the help of fixed movement patterns in which an athlete has to run through a predetermined course as quickly as possible (8). These are movement patterns with closed skills in which there is no reaction to a stimulus. In sports, and especially in sports games, agility movements are usually reactive and require not only physical but also cognitive skills (9, 10).

As a high-intensity intermittent sport, tennis requires players to change direction many times during a match (11). During rallies, players must perform fast, multidirectional movements with constant accelerations, decelerations and changes of direction (CODs) in order to put themselves in the best possible position to return the ball (12, 13). The average number of CODs in tennis varies between 2 and 4 per point (17), depending on the quality of the players and the surface on which a match is played (14, 15).

Various studies have shown that agility is positively related to performance on the court (12–14, 16–20). It is therefore logical to assume that the ability to change the direction of movement quickly plays an important role in achieving peak performance in tennis (21). For this reason, numerous COD tests have been developed and used for tennis over the years. Either generic or more tennis-specific movement patterns were used, in which the athletes had to run through a pre-planned course as quickly as possible (17, 21–25). This type of pre-planned change of direction (COD) test basically measures “players ability to change direction quickly and accurately” (2–5). However, this aspect of agility only covers the “physical” side of agility performance. Recently, however, some reactive agility tests have been developed and used in tennis that involve a response to an external stimulus (16, 19, 20).

Given that the movements in tennis are typically reactive, it is hypothesised that the tests that include a cognitive component of decision making during the fast CODs better represent the type of agility performance required in sports games than the tests in which an athlete must complete a pre-planned course without decision making (9, 10).

However, there is a research gap in the existing sports science literature on tennis, as there are no scientific studies to support this assumption. Therefore, there is a need to determine what type of agility tests can better serve sport scientists and coaches, both in terms of performance assessment of tennis players and feasibility on the field. Furthermore, there is an obvious research gap in obtaining information on the prognostic value of different types of agility tests in predicting the future competitive performance of youth tennis players, as this may be an important asset for talent identification programs given the importance of agility in racquet sports.

The main goal of this study was therefore to determine whether it is possible to successfully differentiate between youth tennis players based solely on the results of different agility tests in relation to their competitive performance. Further aims were to evaluate the psychometric properties of the tests used and to determine whether the type of agility tests used (generic pre-planned CODs/tennis specific pre-planned CODs/tennis specific reactive) makes a difference in predicting the future competitive success of youth tennis players. An initial hypothesis of the authors was that tennis specific agility tests could provide a more reliable and valid assessment of agility performance compared to generic CODs. It was also hypothesised that among tennis-specific tests, the reactive agility test, which incorporates the cognitive response to external stimuli, would prove to be better suited to differentiate between players with different performance levels than the simpler CODs and thus be a better tool to identify talented players in youth categories.

## Methods

### Participants

A total of 33 tennis players, 21 boys and 12 girls (11.05 ± 0.59 years; 152.03 ± 8.56 cm tall; 41.66 ± 6.9 kg) took part in this study. Prior to the study, a sample size estimate was made based on statistical analysis of analogously defined variables in similar studies (26–28), following the guidelines described in Kraemer and Blasey (29). It was concluded that with α = 0.05 and a power of 1-β = 0.80, a sample size of 24–31 was necessary to detect the significant effect of player quality. Consequently, a sample size of 33 young tennis players was used.

As no significant differences (*p* < .05) were found between the genders in the descriptive variables (age, height and weight), the sample was considered homogeneous. All participants were U12 tennis players who competed at national level. To be eligible, they had to have played tennis regularly for at least four years before participating in the study. In general, the participants trained 3 times a week during the first one to one and a half years of their regular tennis training and 5 times a week after this initial training phase. Although the participants could be considered relative beginners in the world of tennis, they had all progressed to the point where they had mastered all the basic strokes and footwork of tennis and were beginning to compete at a national level.

The criteria for determining the performance level of the players were based on a combination of two factors. The first was the Croatian Tennis Association (CTA) ranking for their age group and the second was the expert assessment of the players' performance level by 5 experienced coaches with at least 15 years of experience as coaches in tennis at national level. The expert rating was used in addition to the ranking list points, as the ranking list position in these very young age groups does not always reflect the true performance value of a player. The ranking system is designed to be directly dependent on the number of tournaments played and, for various reasons, not all players participate in the same number of tournaments. In addition to the criteria for the ranking points, the experienced coaches rated the players on the Likert scale and graded them from 1 for the lowest level of performance to 5 for the highest level of performance. All five categories for the classification of the performance level were described in detail in text form in addition to the assigned numerical values, so that the experts had clearly defined criteria for evaluating the performance level of the participants. The fact that the expert coaches already knew the players they were asked to rate also helped to ensure high inter-rater reliability.

Based on the combination of the two criteria, the players were divided into 3 categories according to their performance level:

1. High level players - who achieve remarkable results in their age categories and are in the top 20% of players in their age group.
2. Average level players - who achieve average results in their age categories and are among the 20%–50% of the most successful players in their age group.
3. Low level players - they do not achieve notable results in their age categories and are among the 50% least successful players in their age group.

The study was conducted in accordance with the Declaration of Helsinki, and was approved by the Ethics Committee of the Faculty of Kinesiology University of Split (ID: 2181-205-0205-22-024). As all participants were minors, informed consent was obtained from the parents of all players involved in the study. All participants were informed that they could withdraw from the test at any time without any sanctions.

### Measures/variables

Five validated agility tests were selected for the purpose of this study (20, 30–32).

The selection and number of tests comprising the test battery was based on the following criteria.

Firstly, the test battery should cover three different types of agility tests; generic COD agility, tennis specific COD agility and tennis-specific reactive agility.

Secondly, the selection of tests was based on the feasibility of the tests used and aimed to select validated tests that did not require specialised technical equipment or a long preparation time. This made the test battery practical for use in regular training or testing sessions and facilitated the reproducibility of the results for some future research.

Thirdly, since the tests were conducted at a single time point and each of the 5 tests consisted of three measured trials, participants had to perform 15 different test trials within a relatively short period of time. As all of these trials required maximum effort, the five tests selected were considered the optimal number of trials for the purpose of this study and for this sample of very young U12 players. Based on previous experience with the test battery used (20), the tests were conducted with approximately 3 min of rest between trials and approximately 5 min between tests. The number of tests selected ensured that the participants were able to perform the required test tasks with maximum engagement and maintain their concentration and motivation throughout the test session.

**Figure 1:**
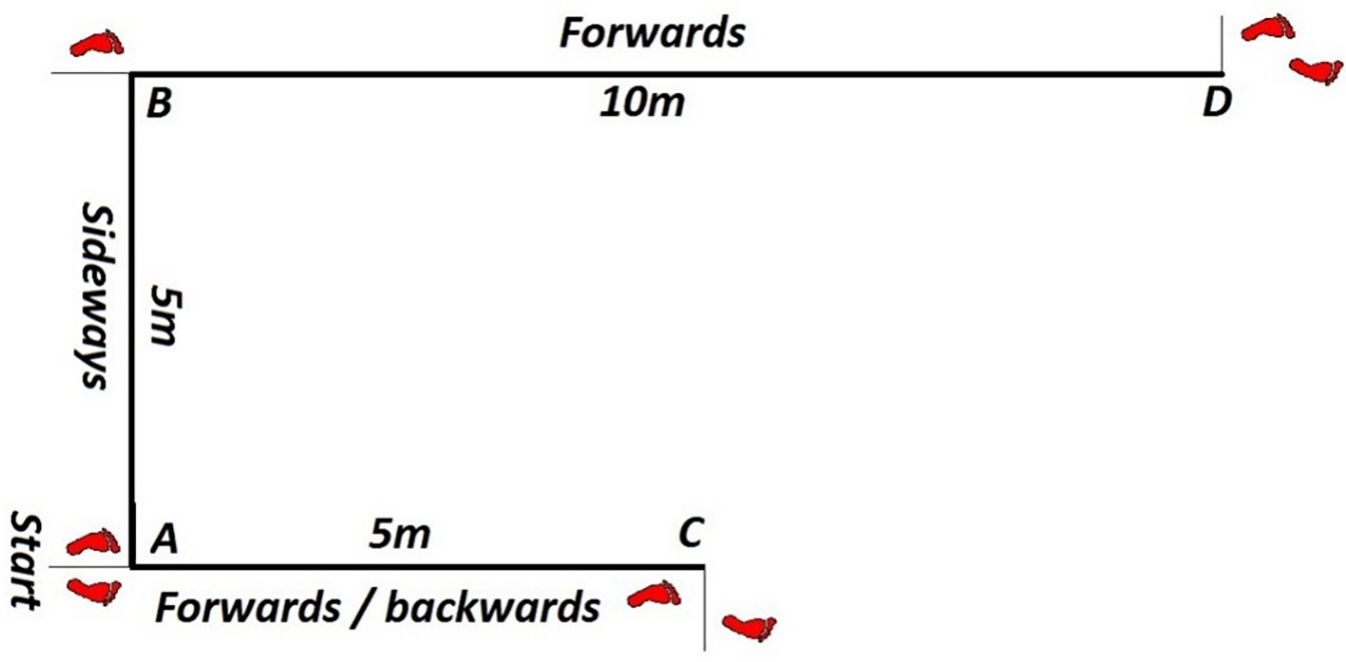
Structure of the multi direction agility test ACABD.

**Figure 2:**
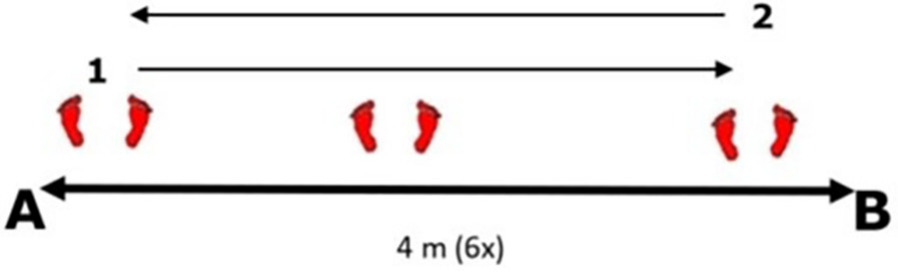
Steps to side – lateral agility test.

**Figure 3:**
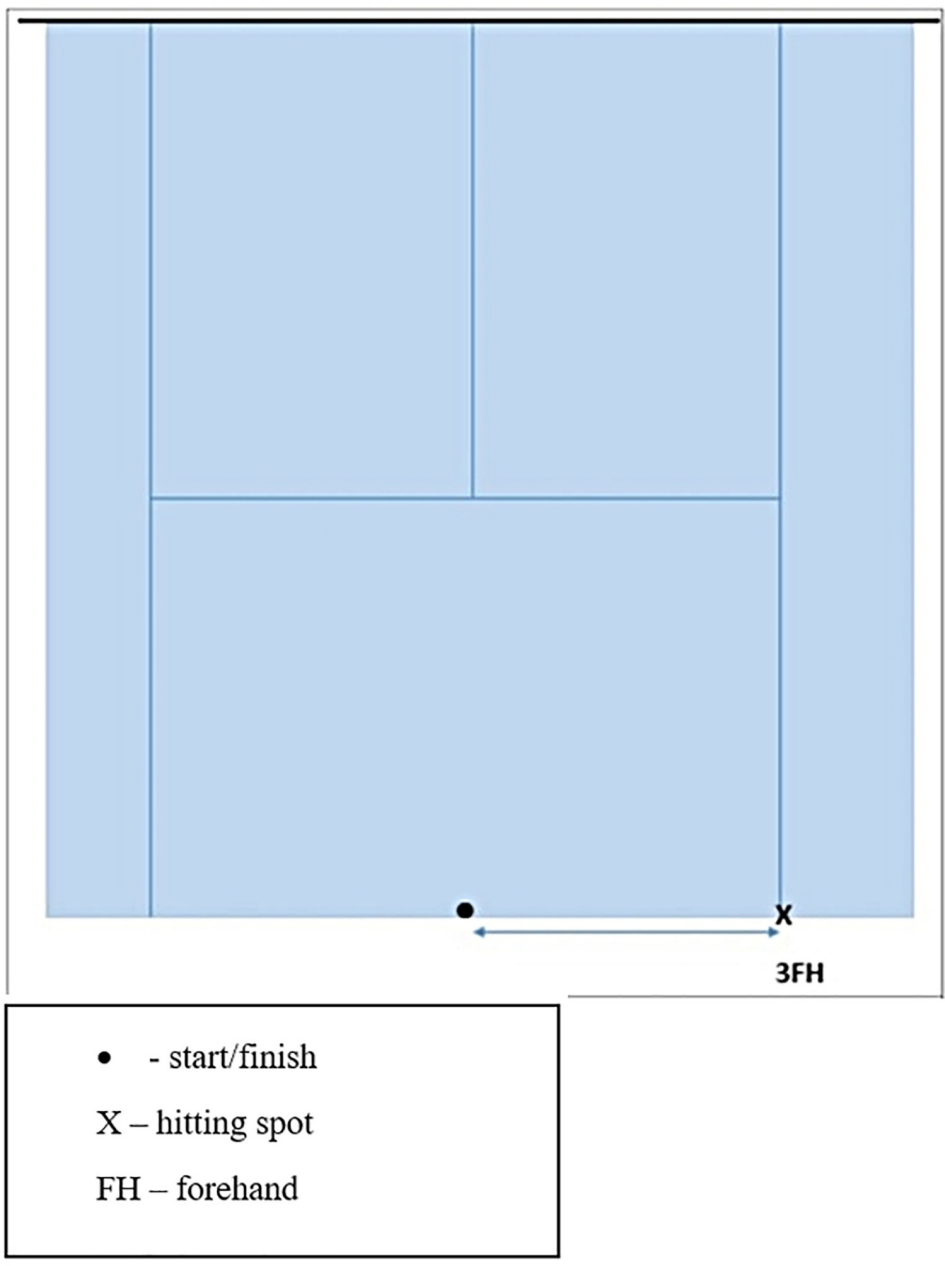
Tennis specific steps to Side lateral agility (TS-STSLA) scheme (22).

**Figure 4:**
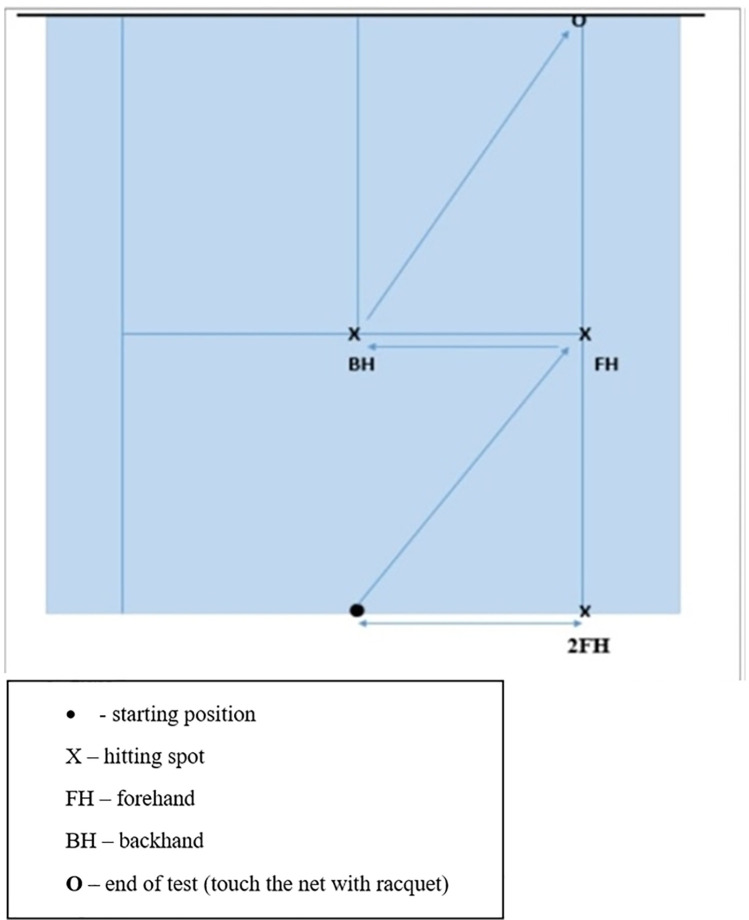
Tennis-Specific multi-directional agility (TS-MDA) test scheme (20).

**Figure 5:**
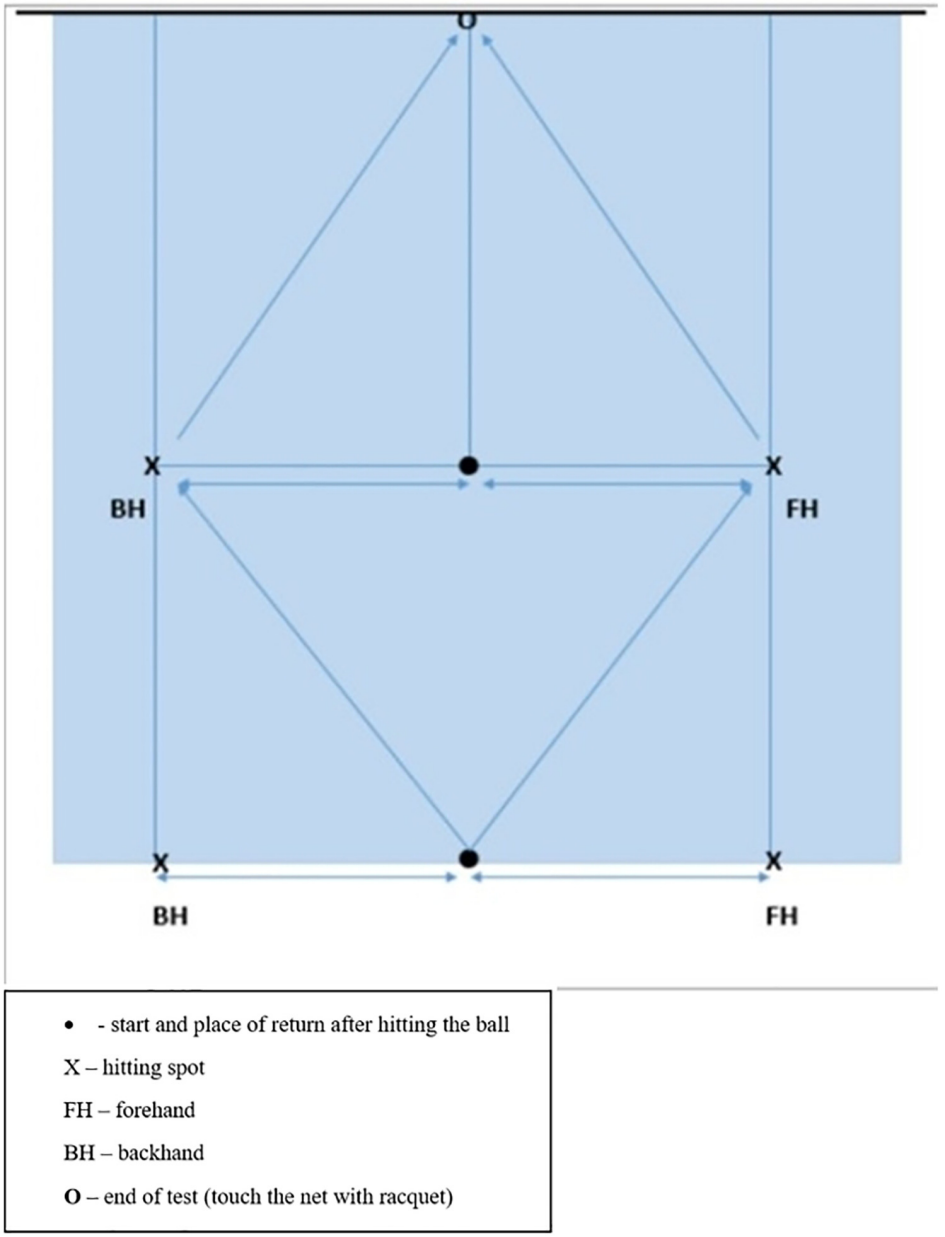
Tennis-Specific reactive agility test (TS-RAT) test scheme (20).

The tests that make up the test battery were as follows:

*Multi Direction Agility “ABCD”* test *(MDA “ABCD”)* is a generic COD agility test (30), very similar in duration and movement pattern (Figure 1) to the other popular and standardly used agility test (T-TEST) (31).
*Steps To Side Lateral Agility (STSLA)* test (32) is a generic agility test that measures the COD ability to move laterally, from left to right and vice versa (Figure 2) The test evaluates lateral speed, agility and body control. The test is almost identical in duration and movement pattern to the commonly used Edgren Side-Step Test (32).
*Tennis-Specific Steps To Side Lateral Agility (TS-STSLA)* test (20) is a test designed to assess the specific lateral COD agility in tennis, which is one of the most common movement patterns of tennis players. The test requires the use of tennis equipment (racket, tennis balls) as players are required to perform a forehand tennis technique on the tennis court. The test is very similar to the generic test “Steps to Side Lateral Agility” (STSLA) test (Figure 3), with the difference that on one side instead of crossing the foot over the line, a forehand technique is performed.
*Tennis-Specific Multi-Directional Agility* (TS-MDA), is a test developed (20) to assess specific multidirectional COD agility in tennis with a movement pattern that simulates the actual situation in the game and require players to execute playing techniques from the marked points on the court (Figure 4).
*Tennis-Specific Reactive Agility Test* (TS-RAT) *i*s a test developed to assess specific multidirectional reactive agility in tennis with a movement pattern that simulates the actual situation in the game, whereby the participants do not know the direction of movement in advance (Figure 5). Therefore, the test includes not only specific multidirectional tennis movements and playing techniques, but also a cognitive response to a visual stimulus.

More detailed information on the tests that make up the test battery can be found in Table 1.

**Table 1:** Agility tests comprising the test battery, categorised according to the type of agility measured and the requirements for the correct performance of the test.

Name of the test (abbreviation)	Type of agility	Space & equipment	Measurement requirements	Task description
Multi-directional agility “ABCD” (MDA “ABCD”)	Generic pre-planned multidirectional COD	Flat open space with hard surface, with a minimum size of 15 × 7 m & Adhesive tape and stopwatch	1 measurer, stands at point D, checks the correct execution of the test and measures the time Time measured in 1/10 of a second/Three measured attempt	The starting position is at point A. The participant covers the distance from A to B and back with sidesteps, sprints forward to point C and backwards to A, sidesteps to point B and concludes with a sprint run to point D
“Steps To Side” lateral agility (STSLA)	Generic pre-planned lateral COD	Flat open space with hard surface, with a minimum size of 5 × 2 m & Adhesive tape and stopwatch	1 measurer, stands in front of the participants, checks the correct execution of the test and measures the time Time measured in 1/10 of a second/Three measured attempt	The starting position is at point A. The participant takes sidesteps to the right until right foot crosses the right outer line at point B and then takes sidesteps back to A. The test is completed when the participant crosses the start/finish line at point A for the 3rd time
Tennis-specific “Steps To Side” lateral agility (TS-STSLA)	Tennis-specific pre-planned lateral COD	Hardcourt tennis court & Stopwatch, 3 tennis balls and tennis racket.	2 measurers. One stands in front of the participants, checks the correct execution of the test and measures the time. The other measurer drops the tennis balls vertically at the intersection of the baseline and the side “singles” line. Time measured in 1/10 of a second/Three measured attempt	The starting position is in the middle of the baseline. The participant moves laterally along the baseline with tennis-specific movements and hits a FH shot at the intersection of the baseline with the right singles’ sideline, and then returns until the left foot crosses the centre of the baseline. The sequence is repeated three times and is completed when the participant crosses the centre of the baseline for the 3rd time
Tennis-specific multi-directional agility (TS-MDA)	Tennis-specific pre-planned multidirectional COD	Hardcourt tennis court & Stopwatch, 4 tennis balls and tennis racket.	3 measurers. One moves along the right singles side-line, checks the correct execution of the test and measures the time. The other two move from the baseline to the service line and drop the balls vertically onto the marked areas. Time measured in 1/10 of a second/Three measured attempt	The starting position is in the middle of the baseline. The participant moves laterally along the baseline with tennis-specific movements and hits a FH shot at the intersection of the baseline with the right “singles” side line, and then returns to the centre of the baseline. This is repeated twice. From the baseline, the participant moves diagonally to the intersection of the service line and the right singles’ sideline, makes a FH shot, moves to the centre of the service line to play the BH shot before sprinting diagonally to the net. The test is completed when the participant's racket touches the marked part of the net.
Tennis-specific reactive agility test (TS-RAT)	Tennis-specific multidirectional reactive agility	Hardcourt tennis court & Stopwatch, 4 tennis balls and tennis racket.	4 measurers. One moves along the singles right side-line, checks the correct execution of the test and measures the time. One gives visual signals by hand indicating movement direction from the opposite side of the net. Two measurers on each side of the lateral singles lines follow the visual signals and drop the balls vertically onto the marked spots. Time measured in 1/10 of a second/Three measured attempt	From the starting position in the middle of the baseline the participant moves to the indicated left/right intersection of the baseline with the right singles’ sideline and hits a FH/BH shot and then returns to the centre of the baseline. This is repeated twice. From the baseline, the participant moves diagonally to the indicated left/right intersection of the service line with the singles’ sidelines, makes a FH/BH shot, moves to the centre of the service line and following the last visual signal plays another FH/BH shot from the left/right intersection of the service line with the singles’ sidelines. The participant then returns to the middle of the service line, sprints to the marked point in the middle of the net and touches it with the racket.

The tests were carried out on a hardcourt outdoor tennis court. The tests were conducted at the same time of day (8:30–10:30 am) at an outdoor temperature of 20°C–25°C. The participants wore the usual tennis clothing worn during training sessions or competitions. All participants brought their own tennis rackets for the tennis-specific agility tests.

### Statistical analysis

The research data were processed with the software package Statistica 14.0.0.15 (TIBCO Software Inc., 2020). Descriptive statistics (mean ± SD) were calculated for all individual trials. The normality of the distributions was checked using the Kolmogorov–Smirnov test with Lilliefors correction. To determine the test-retest reliability of the tests used, the internal consistency between trials was also assessed using Cronbach's alpha (α) and the intraclass correlation coefficient (ICC). The intraclass correlation coefficient (ICC) was interpreted according to the recommendations of Koo and Li (33), with an ICC of <.50 was indicated as poor, between .50 and .75 as moderate, .75–.90 as good, and >.90 being considered excellent.

The degree of agreement between the experts in assessing the players' performance level was determined by analysing various reliability coefficients using both the classical and Guttman methods. The experts were given detailed instructions in text form describing the criteria for each score assigned (1–5). They were also familiar with the players they were to assess from the tournaments and training camps they had previously attended.

To determine the overall dimensionality of the tests used, an exploratory factor analysis using the principal component factor extraction method was performed for all tests forming the test battery and the total explained variance was calculated.

To determine the discriminant validity of the generic and specific agility tests used in relation to the performance level of the young tennis players, a one-way between-subjects ANOVA with a *post hoc* Bonferroni correction was used. The partial eta squared (partial *η*^2^) was used as a measure of effect size. For all calculations, the type I error was set to α = 5%.

## Results

The basic descriptive statistical parameters (M ± SD, distribution of the results) between trials for each of the conducted agility tests are presented in Table 2. The results show a normal data distribution pattern for all the test variables.

**Table 2:** Mean ± standard deviation together with ±95% confidence interval, reliability measures and significance of the kolmogorov–smirnov test with lilliefors correction of the tests used.

Tests	Trial 1	Trial 2	Trial 3	ICC	IIR	Cα	KS test
MDA “ABCD”	11.09 ± 1.15 [10.73–11.45]	10.90 ± 1.21 [10.52–11.27]	10.70 ± 1.14 [10.36–11.04]	.94	0.90	.97	*p* > .10
STSLA	10.02 ± 0.71 [9.82–10.21]	9.86 ± 0.56 [9.70–10.02]	9.80 ± 0.66 [9.60–10.01]	.86	.74	.87	*p* > .20
TS-STSLA	10.07 ± 0.82 [9.84–10.30]	9.71 ± 0.63 [9.53–9.90]	9.43 ± 0.69 [9.21–9.66]	.83	.81	.92	*p* > .20
TS- MDA	12.03 ± 0.72 [11.79–12.27]	11.74 ± 0.76 [11.54–11.93]	11.69 ± 0.85 [11.42–11.95]	.85	.87	.87	*p* > .20
TS-RAT	14.45 ± 1.25 [14.08–14.82]	14.01 ± 1.06 [13.69–14.34]	13.93 ± 1.37 [13.59–14.28]	.89	.80	.91	*p* > .20

[ ], results that lie within 95% confidence interval; ICC, inter class correlation coefficient; IIR, inter-item correlation; Cα, Cronbach alpha.

### Reliability of measurements

The reliability of all agility tests that make up the test battery is shown in Table 2.

Internal reliability measures, the average inter-item correlation (IIR) and Cronbach's alpha (Cα), showed “good” to “excellent” inter-subject reliability. The internal consistency between trials for all agility tests used ranged from (Cα .87–.97) for the Cronbach's alpha coefficient to (IIR .74–.90) for the average inter-item correlation. The intraclass correlation coefficient (ICC) reflects not only the degree of correlation between subjects but also the agreement between measurements (33). The ICC ranged from .83 for the Tennis Specific Steps To Side Lateral Agility (TS-STSLA) test, which is on the edge of “moderate” to “good” test reliability, to .94 for the Multi Direction Agility “ABCD” (MDA “ABCD”) test, which according to Koo and Li (33) is an indicator of “excellent” test reliability.

The generic Multi Direction Agility “ABCD” test (MDA “ABCD”) showed the highest overall reliability, both in terms of internal consistency between trials and agreement between measurements. However, the overall reliability of all tests used shows fairly high and relatively equal values of the different reliability measures across the test battery.

The degree of agreement between the experts in the evaluation of the common object of measurement was determined by analysing various reliability coefficients according to both the classical and Guttman's measurement model (Table 3).

**Table 3:** Determining the level of agreement among the experts in evaluating players’ competitive performance level.

Variable	Cα	*λ*6	h1	msa	V%
Expert grades – (Compet. Perform. Level)	.904	.919	1	.972	66

Cα, Cronbach's coefficient of reliability measured with the classical measuring method on original and standardised results; λ_6_, Guttman-Nicewander's coefficient of reliability measured under Guttman's measurement model; h_1_, homogeneity of the test particles based on the number of principal components with positive coefficients of reliability; msa, Kaiser-Rice's coefficient of the experts’ representation; V_%_, percentage of common variance of the experts’ opinions.

The results presented in Table 3. Show a very high degree of agreement (the measurement reliability is over .90) between the experts and their homogeneity in determining the common object of measurement, regardless of the measurement model used (classical or Guttman). As there was a very high level of agreement among the experts, the expert assessment was considered a valid additional criterion for determining the players' performance level.

### Factor structure and discriminant validity

The results of the exploratory factor analysis showed that a significant latent dimension/factor was extracted that explained 75% of the common variance of the test battery used (Table 4).

**Table 4:** Factor analysis of the variables that make up the test battery for agility.

Variable/Test	Extraction: Principal components
	Factor (F1)
MDA “ABCD”	**.86**
STSLA	**.88**
TS-STSLA	**.85**
TS-MDA	**.88**
TS-RAT	**.85**
Expl.Var	3.73
*Prp.Totl*	.75

Expl. Var., explained variance; Prp.Totl, proportion of total variance explained; Factor (F), correlations of the tests with the main component of factor analysis.

*Values in bold show a significant projection onto the common factor.

All five agility tests that made up the test battery were highly and fairly evenly projected onto the common factor. The correlations of all individual tests with the common latent dimension were in a narrow range between .85 for the TS-STSLA and TS-RAT tests and .88 for the STSLA and TS-MDA tests.

The results showed (Table 5) statistically significant differences in all variables/tests in relation to the performance level of the youth tennis players.

**Table 5:** Differences in test scores, ranking characteristics and basic descriptive parameters between groups of players categorised by performance level, values expressed as M ± SD.

Tests & variables	Competitive performance level			Anova		
	1. High (*n* = 9)	2. Average (*n* = 14)	3. Low (*n* = 10)	F	*p*	*η* ^2^
MDA “ABCD”	10.26^a^ ± 0.95	10.80 ± 0.92	11.60 ± 0.68	5.88	**0.01**	0.28
STSLA	9.65^a^ ± 0.40	9.84 ± 0.48	10.18 ± 0.41	3.53	**0.04**	0.19
TS-STSLA	9.51^a^ ± 0.35	9.52^a^ ± 0.48	10.25 ± 0.51	8.81	**0.01**	0.37
TS- MDA	11.60^a^ ± 0.49	11.62^a^ ± 0.45	12.30 ± 0.59	6.32	**0.01**	0.30
TS-RAT	13.67^a^ ± 0.74	13.92^a^ ± 0.66	14.84 ± 0.93	6.41	**0.01**	0.30
Avg.Rank.	8.67^a^^,^^b^ ± 4.90	43.29^a^ ± 13.81	87.50 ± 11.54	115.37	**<0.01**	0.89
Avg.Rnk.Points	1,628.00^a^^,^^b^ ± 615.04	466.00^a^ ± 175.27	121.90 ± 32.36	51.72	**<0.01**	0.78
Avg.Exp.grade	4.47^a^^,^^b^ ± 0.32	3.63^a^ ± 0.27	2.68 ± 0.30	88.93	**<0.01**	0.86
*Age (years)*	*11.19* ± *0.51*	*11.01* ± *0.65*	*10.96* ± *0.61*	*0*.*38*	*0*.*68*	*0*.*02*
*Height (cm)*	*150.56* ± *9.59*	*153.50* ± *7.61*	*151.30* ± *8.49*	*0*.*39*	*0*.*68*	*0*.*03*
*Weight (kg)*	*39.89* ± *6.79*	*42.79* ± *6.53*	*41.70* ± *7.39*	*0*.*49*	*0*.*62*	*0*.*03*

Avg.Rank., average position on the CTA ranking list; Avg.Rnk.Points, average number of ranking points scored; Avg. Exp.grade, average grade assigned to participants by experts on Likert scale (1–5); F, ANOVA test statistics; *η*^2^, Eta squared, an effect size reported for an ANOVA *F*-test.

^a^ Significant differences from the Low group.

^b^ Significant differences from the Average group.

*Values in bold show significant differences between the groups.

The results also showed that significant differences were found between players with “high” (1st) and “low” (3rd) performance levels in all agility tests used to form the test battery. In addition, the three tennis-specific agility tests (TS-STSLA, TS-MDA, TS-RAT) also revealed significant differences between the “average” (2nd) and “low” (3rd) level players, with players with a higher performance level achieving significantly better results in all tennis-specific agility tests. No statistically significant differences were found between the “high” (1st) and the “average” (2nd) level players in any of the agility tests used, although the mean values of the tests showed slightly higher values in favour of the better-placed players.

## Discussion

Given the importance of agility performance in tennis (13, 16, 18, 34, 35), the main aim of this study was to compare different generic and tennis-specific agility tests, both pre-planned CODs and reactive tests, to determine whether and to what extent they differentiate youth tennis players in terms of their competitive success and can be used to predict their future competitive level.

The data presented in the Results section of the manuscript show that, contrary to the authors' original hypothesis, the different types of agility tests can be considered as equally reliable and valid tools for testing the agility performance of youth tennis players and have similar ability to discriminate between youth players in terms of their competitive performance on the tennis court.

### Reliability of measurements

Reliability is the most important prerequisite for the applicability of the tests. Therefore, the agility tests should show reliable results in order to be able to attribute a difference in the test to a change in the players' performance (19).

In this study the reliability measures, which took into account both intra-subject and inter-subject reliability, showed comparable results for all 3 categories of agility tests (generic COD, tennis-specific COD, tennis-specific reactive). The scores determined by various measures of reliability for all the agility tests used were quite high, ranging from “good” to “excellent”. In comparison, the reliability values obtained were found to be in fairly good agreement with the results of previous research studies (16–22, 24, 25, 34).

The majority of generic agility COD tests that have been widely used in tennis over the years have generally shown good to excellent reproducibility (18, 21, 22, 24, 25). This also applies to the two generic COD agility tests used in this study. The results showed “excellent” reliability both intra and inter subjects for the MDA “ABCD” test and “moderate” to “good” reliability for the “STSLA” test. Both the intra and inter-subject reliability for the two tennis-specific COD agility tests (TS-STSLA, TS-MDA) were also in a similar range to previous studies in which tennis specific COD agility tests were conducted (16, 20, 34).

Regarding the tennis-specific reactive agility tests (TS-RAT), the intraclass correlation coefficient (ICC) showed even higher values (ICC = .89) than in the rare previous studies in which tennis-specific reactive agility tests were performed. The reactive agility test “TAT” used by Jansen et al. (19) showed moderate relative reliability with an ICC of .74 and the same ICC value of .74 was obtained for the reactive agility tests “TS-RAT” (20), which is the same test used in this study.

The results of the various reliability measurements indicate that the different types of agility tests can be regarded as equally reliable instruments for testing the agility performance of tennis players. Regardless of whether the test is generic COD, tennis-specific pre-planned COD or tennis-specific reactive, it can therefore be assumed that it provides coaches with reliable information about the agility performance level of their players.

### Factor structure and discriminant validity of the agility tests used

The exploratory factor analysis showed that only one latent dimension/factor was extracted (Table 4). All five agility tests used from the three test categories showed comparably high projections (from .85 to .88) on a common factor, indicating that their contribution to explaining the common variance is almost identical. These results could point to the conclusion that the different types of agility tests (generic CODs, tennis-specific CODs, tennis-specific reactive) can be considered as equivalent tools for testing the agility performance of tennis players. As all the tests used appear to measure the same construct to a similar degree, sports scientists and coaches should probably opt for the tests that are most feasible and most likely to match the specific aims of their research or training objectives.

The results of one-way analysis of variance (ANOVA) showed (Table 5) that various agility tests can successfully differentiate youth (U12) tennis players in terms of their performance level. Overall, the players who belonged to a higher performance level achieved significantly better results in all agility tests used, regardless of whether the test measured generic COD, tennis-specific COD or tennis-specific reactive agility performance.

Relating the results to those of the few other studies conducted in tennis that examined the ability to discriminate between agility test scores and player performance level, the study by Ulbricht et al. (16) found better performance on the tennis-specific agility test for the more experienced players in the male youth tennis player group (13 years old). In contrast, the study by Ward (36) was unable to differentiate between performance levels on a general agility test conducted with a group of 20-year-old, non-experienced male tennis players. However, studies conducted in other racket sports, particularly badminton, have shown that players with higher performance levels perform better in both specific (37–40) and generic agility tests (41, 42).

When comparing the types of agility tests used (generic CODs/tennis-specific CODs/tennis-specific reactive), it can be seen that all tests within the agility test battery showed a similar ability to discriminate between youth players in terms of their competitive performance on the tennis court. This is in contrast to the findings of Inglis and Bird, who analysed the value and practical applications of reactive agility testing in sport in their systematic review (1). They concluded that reactive agility testing can provide a more reliable and valid assessment of agility performance compared to traditional pre-planned COD agility testing. This was also an initial assumption of the authors of this article, who hypothesised that there would be greater differences in measurement properties between the tennis-specific CODs and the tennis-specific reactive agility tests. The authors also hypothesised that tennis specific CODs might provide a more reliable and valid assessment of agility performance compared to generic CODs However, both hypothesis could not be confirmed in the case of this study and for this sample of youth (U12) tennis players. The three tennis-specific agility tests were found to be slightly more sensitive in discriminating between “average” (2nd) and the “low” (3rd) level players, but overall, the reliability, factorial and discriminant validity of all the tests used within the agility test battery showed fairly equal values.

The authors considered that the age of the players could be one of the possible reasons why the tennis-specific reactive agility test did not provide a more reliable and valid assessment of agility performance than the traditional, generic. COD agility tests. The age of the participants, who belong to the U12 category, could be an important factor in interpreting the results of the study. At this young age, the technical-tactical development of the players is still in full swing and it is quite possible that the generic agility tests are more suitable for the coaches only until the players have reached a certain age and technical level.

Furthermore, players at a young developmental age who are biologically advanced often achieve better results than other players who are technically more solid but have undergone late development. Therefore, the influence of the physical component on athletic performance may be more strongly emphasised at these developmental ages (43). It should therefore come as no surprise that the basic generic agility COD tests, which emphasise motor skills, may serve as good or even better predictors of performance levels in youth players than the more technically demanding tennis-specific reactive agility tests. In the case of this study, however, there is no indication that the developmental factors are the reason why the tennis-specific tests do not perform better than the generic agility tests, as no significant differences were found between the three categories of performance level with regard to the chronological age, height and weight of the players (Table 5). So, this can only be regarded as an assumption on the part of the authors.

### Practical implications

Although many studies have been conducted over the years to evaluate the agility performance of tennis players, they have mainly focused on the reliability and criterion validity of the tests used (18, 20–22, 24, 25, 34, etc.). Only in the studies by Ulbricht et al. (16) and Jansen et al. (19) were the results of agility tests linked to a criterion for competition results. Furthermore, none of the studies conducted compared the different types of agility tests (generic CODs/tennis-specific CODs/tennis-specific reactive) and related them to the performance level of tennis players. The results of this study suggest that both generic and tennis-specific agility tests, whether pre-planned COD or reactive, are equivalent in terms of reliability and ability to discriminate between youth players in terms of their performance level.

The results obtained raise the question of whether it is necessary to use more complex, sport-specific tests as opposed to simpler, generic tests to assess the performance and potential of youth tennis players. Therefore, if these results are confirmed in future studies conducted in different age groups and at different performance levels, it would probably be legitimate for coaches to opt for the simpler and generally more feasible generic tests. However, if the tests have similar metric properties and prognostic value, the final choice of agility tests to be used in a particular case should primarily depend on the specific goals and training objectives of the coach in question.

The results of the study show that various agility tests, whether simpler CODs or reactive ones, can be a useful tool for coaches in identifying talent. Considering the complexity of the game of tennis, this should of course be done together with some other measurable indicators of the players' anthropological and technical-tactical status and potential. As all the tests that make up the test battery are very feasible and can easily be administered during regular training or testing sessions, they can also be used to regularly assess agility performance and monitor players' progress.

### Limitations of the study

The sample only relates to a specific, young age group (U12). Future research studies should look at players of different ages and performance levels to determine the influence of different agility tests on the prediction of future tennis performance. They should also investigate whether tennis-specific reactive agility tests are more meaningful in older, more mature athletes.

Another limitation of the study is the manual timing of the participants' results. In selecting the agility tests, the authors opted for feasibility and reproducibility rather than tests with more sophisticated technical equipment, such as photocells and lighting systems. Of course, the use of electronic timing systems is always an added bonus that reduces measurement errors. However, manual timing is still a viable method for coaches who do not have access to expensive equipment. In addition, three trials were conducted for each test to minimise measurement error.

Finally, no calculations were made in this study to determine the biological age of the participants, as no significant differences were found between the three categories of performance level in terms of chronological age, height and weight of the participants. However, the use of methods to determine the biological age of youth participants, such as peak height velocity (PHV) prediction, may provide additional insight into the characteristics of the study sample, as the biological age of participants can and does influence the performance of players in youth categories (43). Therefore, if the sample consists of participants at a sensitive developmental age, it is a recommendation for future studies to include maturation calculations in the research methods.

## Conclusion

The results of the study suggest that the different agility tests have the potential to differentiate between different quality levels of youth tennis players, regardless of which type of test (generic COD/tennis-specific COD/tennis-specific reactive) is used. Although tennis is an extremely complex sport game with many factors influencing players' competitive performance, different agility tests can be considered as useful tools for predicting the future performance level of youth tennis players.

The results of the study could be important for tennis coaches as they emphasize the importance of agility performance for achieving competitive results in tennis. They also suggest that various agility tests can be used by coaches to identify talented players in youth categories. Furthermore, the results suggest that simpler generic agility COD tests could provide coaches with equally relevant information as the more complex tennis-specific tests. Considering that the tests were carried out in a young age group (U12), the results obtained could also indicate that generic agility COD tests are better or at least equally suitable for coaches, until the players have reached a certain age and technical skill level.

Future studies, on different age groups and performance levels are certainly needed to further determine the importance and role of different agility tests in assessing the performance and potential of youth tennis players.

## Data Availability

The raw data supporting the conclusions of this article will be made available by the authors, without undue reservation.
